# NF-*κ*B Inhibitory Activity of the Di-Hydroxy Derivative of Piperlongumine (PL-18)

**DOI:** 10.1155/jimr/9915695

**Published:** 2025-01-08

**Authors:** Yael Schlichter Kadosh, Subramani Muthuraman, Ariel Kushmaro, Rajendran Saravana Kumar, Jacob Gopas

**Affiliations:** ^1^Avram and Stella Goldstein-Goren Department of Biotechnology Engineering, Ben Gurion University of the Negev, Beer Sheva 84105, Israel; ^2^Department of Chemistry, Vellore Institute of Technology, Chennai Campus, Chennai 600127, Tamilnadu, India; ^3^The Ilse Katz Center for Nanoscale Science and Technology, Ben Gurion University of the Negev, Beer Sheva 84105, Israel; ^4^School of Sustainability and Climate Change, Ben Gurion University of the Negev, Beer Sheva 84105, Israel; ^5^Department of Microbiology, Immunology and Genetics Faculty of Health Sciences, Ben Gurion University of the Negev, Beer Sheva 84105, Israel

## Abstract

Inflammation is a critical response of the immune system to infection or injury, serving to repair and restore tissue homeostasis. While acute inflammation generally protects against harmful stimuli, prolonged and chronic inflammation have detrimental effects and disrupts tissue homeostasis. Due to the complex and multifactorial etiology of chronic inflammation, effective treatment remains elusive. We found that piperlongumine (PL)-18, a di-hydroxy derivative of PL from long pepper, inhibits the nuclear factor kappa B (NF-*k*B), a master transcription factor of numerous components of the inflammatory response. NF-*k*B was inhibited by PL-18 in two human cell-lines, L428 and A549, by preventing the nuclear translocation of p65 NF-*k*B. We also found that I*κ*B kinase (IKK) was degraded in the presence of PL-18. Furthermore, PL-18 inhibited the production of proinflammatory cytokines expressed by L428, a cell line with a constitutive active NF-*k*B. Altogether, our results suggest that PL-18 is a molecule of interest to be further developed to treat persistent infections with severe inflammation.

## 1. Introduction

Inflammation is a stress response to various stimuli (e.g., injury, infection, and tissue stress). The inflammatory responses are tightly regulated so that the inflammation can be balanced between the elimination of the insult and possible damage to the tissues [1, 2]. An imbalance in the regulation of inflammation may result in increased morbidity and mortality [3].

Today, anti-inflammatory drugs are either nonsteroidal anti-inflammatory drugs (NSAIDs) or steroid-based agents [4–10]. Many of them with moderate to severe and long-term adverse effects [11–13]. Therefore, there is a need for new effective agents to modulate inflammation processes with minimal adverse side effects.

Nuclear factor kappa B (NF-*k*B) is a major transcription factor that regulates many processes, including inflammation [14], and thus could be a target for inhibition. NF-*k*B plays an important role in first-line protection against pathogens (e.g., viruses or bacteria) and is evolutionarily conserved [15]. Consequently, some pathogens evolved to specifically modulate the NF-*k*B pathway [16]. The NF-*κ*B family of transcription factors includes five members, RelA (p65)-p50, relB-p52, and c-Rel, acting as dimers. The Inactive form of NF-*κ*B is complexed with its direct inhibitor (I*κ*B) in the cytoplasm. Many stimuli may induce NF-*κ*B activation, including tumor necrosis factor *α* (TNF*α*), interleukin 1 (IL-1), bacterial lipopolysaccharide (LPS), viral double-stranded (ds) RNA, and ionizing radiation. In response to these and other stimuli, I*κ*B is phosphorylated by I*κ*B kinase (IKK), then ubiquitinated and degraded, releasing the NF-*κ*B dimer to translocate to the nucleus in order to activate the inflammatory response [17, 18].

Plant secondary metabolites have long been considered an important source for drug discovery. Many plant base molecules can modulate NF-*κ*B, including curcumin from *Curcuma longa*, nupharidines from *Nuphar lutea*, *and* Resveratrol from *Vitis vinifera* [14, 19]. The synthesis of new derivatives of these metabolites increases the probability of finding new drugs for many therapeutic purposes. Piperlongumine (PL) is an amide alkaloid isolated from long pepper (*Piper longum*). The synthesis of its derivatives has been described in our previous works [20, 21]. PL has multiple biological activities [22] but is mainly known as an anticancer agent that selectively inhibits cancerous cell lines [23, 24]. PL was shown to directly inhibit the NF-*κ*B pathway by interacting with IKK and elevation of reactive oxygen species (ROS) [25]. It also moderately inhibits bacterial and fungal growth [26]. In this study, the synthetic di-hydroxyl derivative of PL, PL-18, was shown to effectively inhibit NF-*k*B activation. Previously, we have found that PL-18 significantly inhibits bacterial quorum sensing (QS) based communication, reduces biofilm biomass [27], has antioxidant activity, and in contrast to PL, PL-18 is less cytotoxic to the tested cell-lines [20]. Thus, the ability to simultaneously inhibit NF-*κ*B mediated inflammation and bacterial communication provides the opportunity to develop new dual and effective molecules against persistent bacterial infection and inflammation.

## 2. Materials and Methods

### 2.1. PL-18 Source

**Figure 1:**
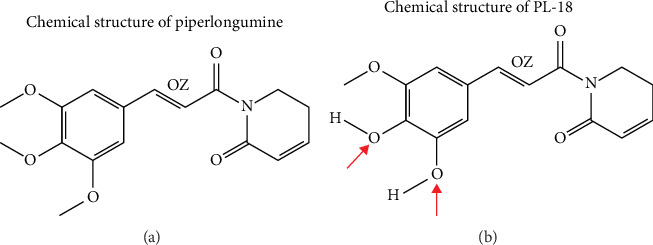
PL and PL-18 chemical structure: (A) PL and (B) PL-18. PL, piperlongumine.

PL required for the present study was isolated from long pepper; it was sequentially demethylated to obtain dihydroxy PL (PL-18), as previously described by Subramani et al. [20] and Muthuraman et al. [28] (Figure 1).

### 2.2. Viability of L428 Cells Using Trypan Blue

The human Hodgkin's Lymphoma L428 cell line was used as a model. The cells were grown in RPMI Medium 1640 with 10% FBS, 1% glutamine, and pen-strep (all from Biological Industries, Beit-Hemed, Israel). In total, 200,000 cells/well were placed in 96-well plates with PL-18 for different times and concentrations (Figure 1A,B). The incubations were followed by the addition of trypan blue (Biological Industries) at a 1:1 ratio. The counting and recording of live cells were done using a hemocytometer.

### 2.3. NF-*k*B-Luciferase Reporter Gene Assay

The effect of PL-18 on NF-*k*B was determined on L428 cells stably transfected with the luciferase NF-*k*B-Luc reporter gene, as described in Ozer et al. [29] and Kadosh et al. [30]. The cells lack I*κ*B, therefore NF-*k*B is constitutively active. 10^6^ cells/well in triplicates were incubated for 2 h in 1 mL of medium containing the solvent (DMSO) or different concentrations of PL-18. Cells were then harvested, lysed, and monitored by a luciferase reporter assay kit (Promega), according to the manufacturer's instructions. Data were normalized to the protein concentration in each lysate as measured by the Bradford method (Bio-Rad).

### 2.4. Time and Dose Response of p65 NF-*k*B Activation of TNF*α* -Stimulated A549 Cells

To further test the effect of PL-18 on NF-*k*B activation, the A549 adherent human lung epithelial cells were used. These cells have an intact NF-*k*B signaling pathway. Immunofluorescence experiments were performed as described before [30]. After different treatments, the cells were washed, fixated, permeabilized, and blocked before immunostaining with primary antibody anti-p65 (mouse anti-p65 [F-6] SC-8008, Santa Cruz, USA). Then, with a secondary antibody AF488 (green) (Goat anti-Mouse IgG [H + L] Alexa Fluor 488 [A-11029] Invitrogen, Thermo Fisher Scientific, Ma, USA). As well as nuclear staining with DAPI. Cell fluorescence was imaged and quantitated by the Operetta High-Content Imaging System (Perkin Elmer) at 40× magnification. Analysis of the images was done through the Columbus server of the company, where parameters can be defined, such as cell area, nucleus, and cytoplasm, separately. The mean nuclear/cytoplasm fluorescence intensity ratio values were determined for each well.

### 2.5. Western Blot (WB) Analysis of IKK in L428 and A549 Cell-Lines

To determine the IKK response to PL-18, WB was performed. L428 or A549 cells were incubated with two PL-18 concentrations (40 and 160 µM). A549 cells were incubated with or without TNF*α*. The cells were then washed twice with cold PBS and lysed with RIPA lysis buffer containing protease cocktail and phosphatase inhibitors and incubated on ice for 30 min. Then, the lysate was passed 10 times through a 21G needle and centrifuged (30,000 RPM at 4°C for 30 min). Protein concentration was measured by Bradford assay, and 15 µg protein was mixed with sample buffer (GenScript, A_2_S technologies, Yavne, Israel) and boiled at 95°C for 5 min. Then, samples were loaded to a 10% acryl amide (37.5:1 acrylamide/bisacrylamide, Bio-Lab, Israel) SDS–PAGE. Proteins were transferred to a nitrocellulose membrane, blocked with 5% Bovine Serum Albumin (BSA, Roth, A_2_S technologies) for 1 h at room temperature (RT), washed with TBST before overnight incubation with the primary antibody, anti-IKK (mouse anti-IKK alpha, VMA00667, Bio-Rad) at 4°C, washed again, and incubated with a secondary antibody peroxidase-conjugated anti-mouse (Peroxidase AffiniPure Donkey Anti-Mouse IgG [H + L], Jackson ImmunoResearch, USA). Chemiluminescence was detected by gel imaging (Azure Biosystems 400, CA, USA). The actin (Monoclonal Anti-b-Actin-Peroxidase: Sigma-Aldrich, MO, USA) levels were determined as well. Bands quantification was performed by ImageJ.

### 2.6. Cytokine Quantification

To test the effect of PL-18 on cytokines secretion, we used L428 cells and measured the presence of cytokines in the supernatant using the human proteome cytokine array (Proteome Profiler Human Cytokine Array, ARY005b, R&D systems). L428 Cells were centrifuged at 1000 rpm for 5 min, and the supernatant was discarded. The pellet was washed twice by adding PBS, centrifuged, and then discarded. Fresh and supplemented RPMI medium was added to a final concentration of 5X10^5^ cells/mL (1X10^6^ cells per well, 2 mL final volume) in triplicates. DMSO or 160 µM PL-18 (dissolved in DMSO) were added. The cells were then incubated for 4 h at 37°C, 5% CO_2_. After, the supernatant was collected by centrifugation. Then, 600 µL of the supernatant was immediately applied to the human proteome cytokines array according to the manufacturer's instructions. Determination of the spots' density was performed by ImageJ.

### 2.7. Statistical Analysis

All statistical analyses were carried out using GraphPad Prism version 8.0.1 for Windows (GraphPad Software, San Diego, California USA, www.graphpad.com). The specific tests are indicated under the relevant figures.

## 3. Results

### 3.1. Determination of PL-18 Cytotoxicity on L428 Cells

**Figure 2:**
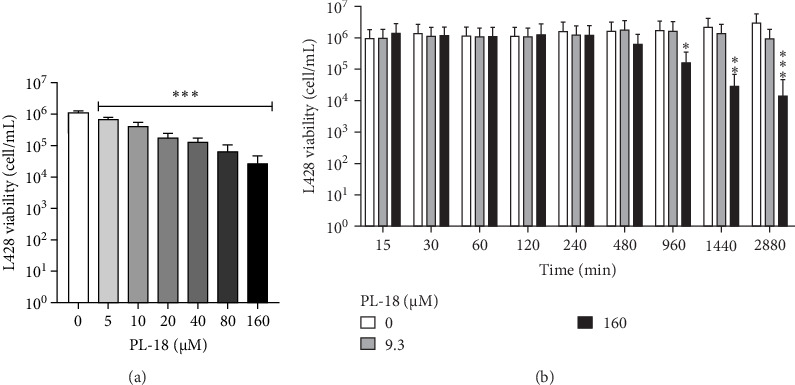
The effect of PL-18 on L428 cell viability. (A) L428 cells were incubated with increasing concentrations of PL-18 or with vehicle (DMSO) for 48 h. Cell viability was determined by trypan-blue exclusion. One-way ANOVA, Dunnett's multiple comparison correction tests with 95% confidence interval, mean + SD, *N* = 6 in two independent experiments. (B) The cells were incubated with two concentrations of PL-18 (LC_50_−9.3 µM and 160 µM) or vehicle for several time points. The graph shows mean ± SD, two-way ANOVA, 95% confidence interval (*p*-values: *⁣*^*∗*^≤ 0.0332, *⁣*^*∗∗*^≤ 0.0021, and *⁣*^*∗∗∗*^≤ 0.0002). *N* = 6 in two independent experiments. PL, piperlongumine.

To determine the nontoxic conditions of PL-18 required for NF-*k*B inhibition, L428 cells were incubated with PL-18 at several time points and concentrations. In Figure 2A,B, we showed that PL-18 becomes toxic after 16 h at 160 µM and that the LC_50_ concentration after 48 h is 9.3 µM (LC_50_ calculation is presented in the Figure S1).

### 3.2. PL-18 Inhibits NF-*k*B Activation in L428 Cells

**Figure 3:**
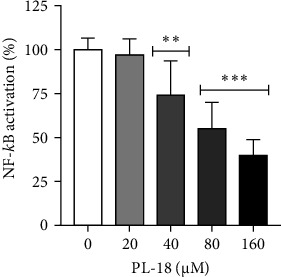
The effect of PL-18 on NF-*k*B activation. L428 cells were stably transfected with the NF-*κ*B luciferase reporter gene. The cells were incubated with PL-18 at different concentrations for 2 h. The cells were lysed, and luminescence was determined. The results represent the percentage of NF-*κ*B activation with PL-18 as compared to vehicle (DMSO) treated cells. All samples were normalized to the lysate protein concentration. Mean + SD, *N* = 9 in three independent experiments. One-way ANOVA and Dunnett's multiple comparison test. 95% confidence interval (*p*-values *⁣*^*∗∗*^≤ 0.0021 and *⁣*^*∗∗∗*^≤ 0.0002). NF-*k*B, nuclear factor kappa B; PL, piperlongumine.

The ability of PL-18 to inhibit NF-*κ*B activation was tested in L428 cells where NF-*κ*B is constitutively active due to lack of I*κ*B [29]. It significantly inhibited NF-*k*B in L428 cells in a dose-dependent manner. Incubation of L428 cells with 160 µM PL-18 for 2 h resulted in 60% NF-*k*B inhibition (Figure 3).

### 3.3. PL-18 Inhibits NF-*k*B Activation in A549 Cells

**Figure 4:**
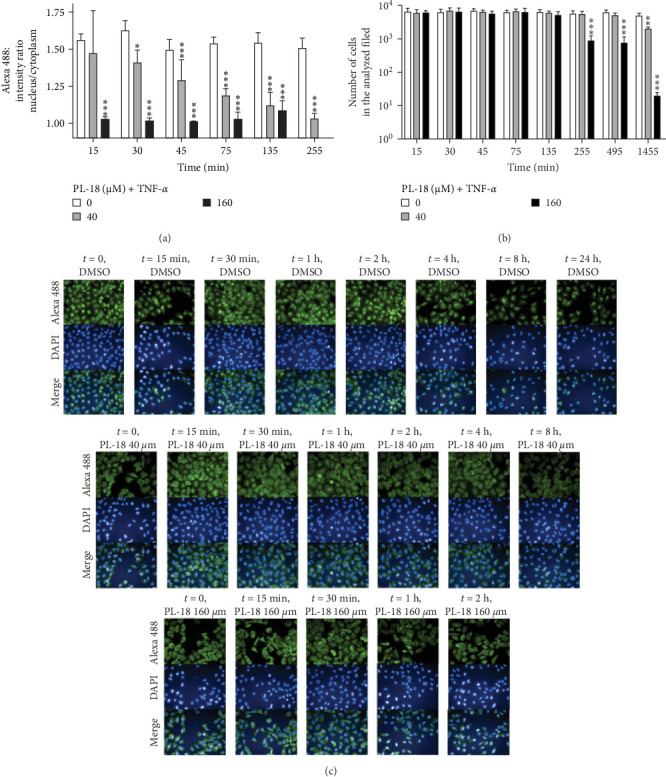
PL-18 inhibits NF-*k*B activation in a time-dependent manner. (A) A549 cells were coincubated with two concentrations of PL-18 or vehicle (DMSO) at different times. NF-*k*B was activated with 2.5 ng/mL TNF*α* for 15 min before the cells were fixed and immunostained. The ratio of the mean nuclear/cytoplasm fluorescence intensity values of 30 fields is shown. A ratio above 1 represents increased NF-*κ*B activation. Mean + SD, *N* = 4 in two independent experiments. Two-way ANOVA, Dunnett's multiple comparison test. (B) The graph represents the number of cells from the analyzed fields. Two-way ANOVA, Tukey's multiple comparison test (*p*-values: *⁣*^*∗*^≤ 0.0332, *⁣*^*∗∗*^≤ 0.0021, and *⁣*^*∗∗∗*^≤ 0.0002). (C) Representative Operetta fluorescent images, *t* = time (h). For treated A549 cells (A, B, and C), the cells were fixed in paraformaldehyde and immunostained. The nucleus was stained with DAPI (blue) and p65 with mouse-anti p65 and Alexa 488 (green) fluorescent goat antimouse IgG. Fluorescence microscopy was performed by the Operetta imaging system, and the analysis at the single-cell level was performed by the Columbus software. NF-*k*B, nuclear factor kappa B; PL, piperlongumine; TNFα, tumor necrosis factor *α*.

**Figure 5:**
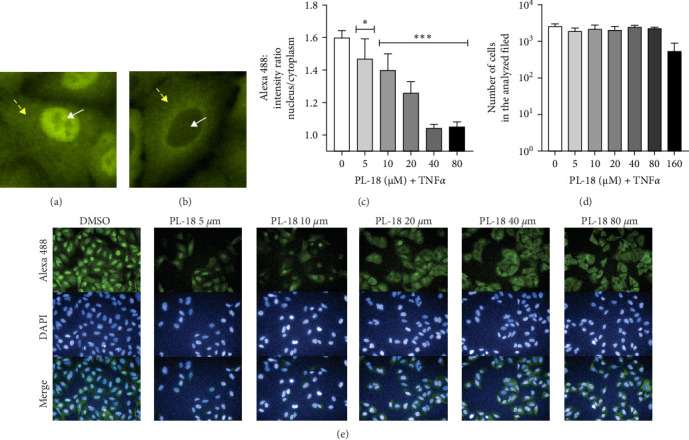
PL-18 dose–response inhibition of NF-*k*B activation. A549 cells were fixed and immunostained with mouse-anti p65 and Alexa 488 fluorescent goat anti-mouse IgG and with DAPI. Fluorescence microscopy analysis was performed by the Operetta imaging system and the Columbus software, the same as in Figure 2. (A) NF-*k*B was activated with 2.5 ng/mL TNF*α* for 15 min, and p65 was detected in the nucleus. (B) Untreated A549 cells-inactive p65 in the cytoplasm. Yellow arrows point to the cytoplasm, and white arrows point to the nucleus. (C) A549 cells were coincubated with various PL-18 concentrations or DMSO for 4 h. NF-*k*B was then activated with 2.5 ng/mL TNF*α* for 15 min before fixation and immunostaining. In the graph, the ratio of the mean nuclear/cytoplasm fluorescence intensity values in the analyzed fields from 30 fields is shown. (D) The graph presents the number of cells in the analyzed fields. Mean ± SD, *N* = 6 in three independent experiments. One-way ANOVA, Dunnett's multiple comparison test. (E) Representative Operetta fluorescent images. *p*-values: *⁣*^*∗*^≤ 0.0332 and *⁣*^*∗∗∗*^≤ 0.0002. NF-*k*B, nuclear factor kappa B; PL, piperlongumine; TNF*α*, tumor necrosis factor *α*.

We also tested NF-*k*B inhibition in a second cell line, A549, where, in contrast to L428 cells, the NF-*k*B pathway is intact and was activated by TNF*α*. PL-18 at 160 µM after 2 h incubation inhibited NF-*k*B activation by 84% in TNF*α* activated cells (Figure 4A,C). At 40 µM, the time response was effective but more gradual, with the best inhibitory activity at 4 h (Figure 4B,C). Therefore, the dose–response experiments in Figure 4 were performed at 4 h. These experiments showed that PL-18 inhibits NF-*k*B activation in a dose-dependent manner (Figure 5C–E). The activation of NF-*k*B is defined as the translocation of p65 from the cytosol to the nucleus. The NF-*k*B (p65) fluorescence intensity ratio between the nucleus and the cytoplasm of A549 cells was determined. A ratio of 1 is the baseline (no activation), and a ratio of >1 represents activation (Figure 5A,B). In these experiments, we also showed the number of cells per analyzed field, which indicates the cytotoxic effect of PL-18 on A549. The toxicity at 160 µM begins at 4 h. The results presented regarding NF-*k*B activation were performed at noncytotoxic conditions (Figures 4B and 5D). The total cellular fluorescence intensity (cytoplasm + nucleus) did not change in response to PL-18 at different incubation times and concentrations. This indicates that NF-*k*B was not due to p65 degradation (Figure S2A,B).

**Figure 6:**
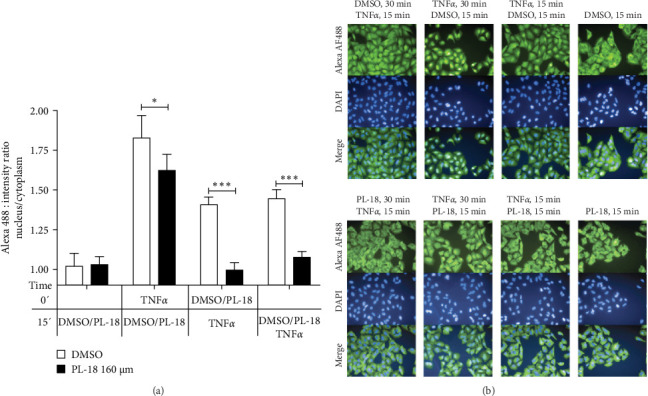
PL-18 inhibits NF-*k*B activation both before, together, and after the addition of TNF*α*. (A) Control A549 cells were coincubated with DMSO with or without TNF*α* at different times. To determine the effectiveness of PL-18, it was added before, together, or after TNF*α*, as shown in the graph. 15 min after incubation with all the treatments (30 min from time 0), the cells were fixed and immunostained with mouse-anti p65 and fluorescent goat anti-mouse IgG and with DAPI. Fluorescence microscopy analysis was performed using the Operetta imaging system and the Columbus software. The mean nuclear/cytoplasm fluorescence intensity ratio values were determined in 120 fields in each well. Mean ± SD, *N* = 5 in three independent experiments is presented. An unpaired, two-tailed *t*-test was performed for each data pair (DMSO/PL-18), with a 95% confidence interval (*p*-values: *⁣*^*∗*^≤ 0.0332 and *⁣*^*∗∗∗*^≤ 0.0002). (B) Representative Operetta fluorescent images. NF-*k*B, nuclear factor kappa B; PL, piperlongumine; TNF*α*, tumor necrosis factor *α*.

To further understand the stage where it is most effective, PL-18 was added either before, together, or after the addition of TNF*α*. The results in Figure 6A showed that the addition of PL-18 before or together with TNF*α* resulted in optimal inhibition of NF-*k*B activation. Its addition following TNF*α* was much less effective (Figure 6A,B).

### 3.4. IKK Response to PL-18 in A549 and L428 Cells

**Figure 7:**
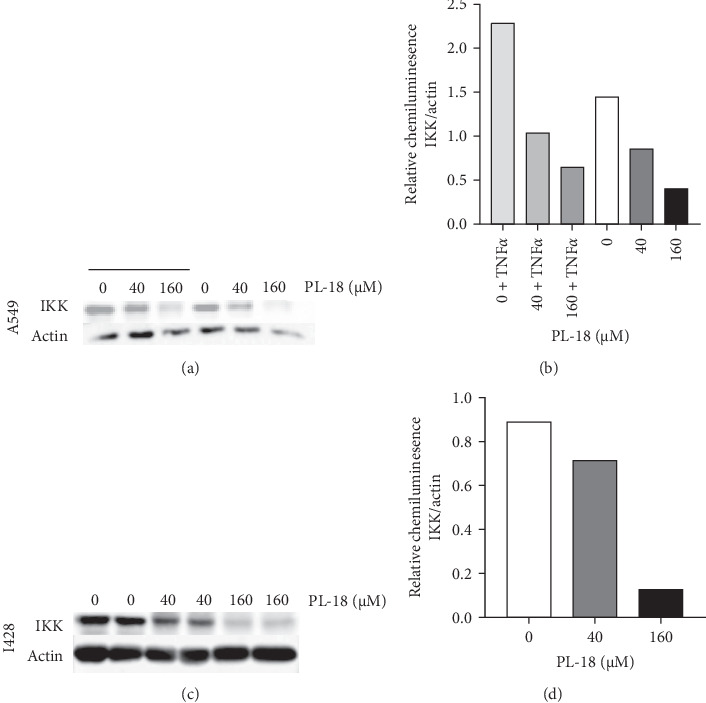
PL-18 decreases IKK amounts in A549 and L428 cells. (A and B) L428 cells were incubated with two concentrations of PL-18 or DMSO (0) in duplicates for 2 h, (D and C) A549 cells were incubated with two concentrations of PL-18 or DMSO (0) for 2 h, with or without TNF*α* 2.5 ng/mL for 15 min. Both cell-lines were then lysed and analyzed by western blot. IKK was detected with mouse anti-IKK followed by peroxidase-conjugated anti-mouse IgG. Actin was detected with peroxidase-conjugated antiactin alpha. Bands quantification was performed by ImageJ. The graphs showed the ratio between IKK and actin bands. IKK, I*κ*B kinase; PL, piperlongumine; TNF*α*, tumor necrosis factor *α*.

Since IKK is an upstream central modulator of the NF-*k*B pathway, we asked whether PL-18 affects its expression in A549 and L428 cells. The results in both cell-lines showed similar results; regardless of TNF*α* activation, IKK presence in the cells is decreased when PL-18 is added in a dose-dependent manner (Figure 7A–D).

### 3.5. PL-18 Reduces Cytokine Secretion by L428 Cells

**Figure 8:**
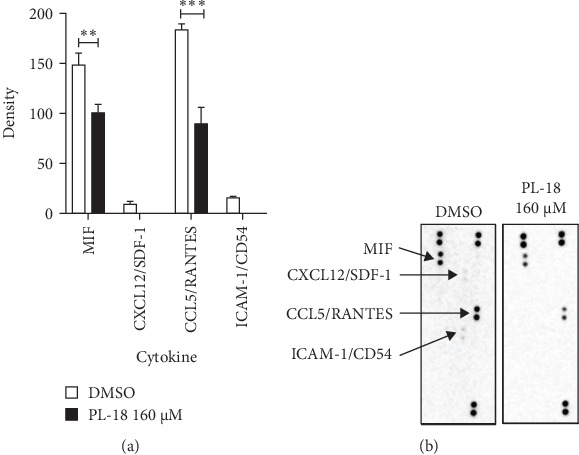
Cytokines quantification in L428 cells supernatant exposed to PL-18. The L428 cells were treated with 160 µM PL-18 or DMSO for 4 h. Then, the supernatants were collected and applied to the human proteome cytokines array. The developed membrane was analyzed by ImageJ. (A) spots quantification, the graph presents the mean of duplicate spots after subtracting the blank (background) and analyzed by two-way ANOVA, followed by Sidak's multiple comparisons test (*p*-values: *⁣*^*∗∗*^≤ 0.0021, *⁣*^*∗∗∗*^≤ 0.0002). (B) In the image of the cytokines array membranes, the reference spots are unmarked, and cytokines spots are marked with arrows. CCL5, chemokine C-C motif ligand 5; CXCL12/SDF-1, stromal cell-derived factor 1, also known as C-X-C motif chemokine 12; ICAM-1, intercellular adhesion molecule 1; MIF, migration inhibitory factor; PL, piperlongumine.

NF-*k*B inhibition by PL-18 is expected to cause a reduction in the secretion of cytokines. To this end, we measured cytokines' presence in the supernatant of L428 cells using a proteome human cytokines array. The Table S1 and Figure 3B present a long exposure time membrane as this exposure time reveals more cytokines than the short exposure time but was less quantifiable. Thus, the density of the spots was determined on the short exposure time, as presented in Figure 8A,B. Four duplicate spots of cytokines are shown: MIF (migration inhibitory factor), CCL5 (chemokine C-C motif ligand 5)/RANTES, ICAM-1 (intercellular adhesion molecule 1)/CD54, and CXCL12/SDF-1 (stromal cell-derived factor 1, also known as C-X-C motif chemokine 12) were detected in the DMSO control group. The four spots were downregulated in the PL-18-treated supernatant, and two were undetectable. Thus, PL-18 reduces these four cytokines' secretion, supporting its NF-*k*B inhibitor role.

## 4. Discussion

Inflammation attenuation is an important part of many medical conditions, such as chronic inflammatory diseases, allergies, and various infections. Today, anti-inflammatory drugs (e.g., steroid-based medication) have multiple side effects; some are severe and long-term. Several medical situations that are characterized by persistent infection and inflammation are treated with both antibiotics and steroids to address both aspects of the conditions. For example, postsurgery infections [31], chronic wound infections [32], and oral implants-associated infections [7, 33]. Steroid-based medication is associated with reduced swelling and pain and increased patient comfort [34]. Since we have previously shown that PL has anti-inflammatory properties and its derivative, PL-18, is an effective inhibitor of bacterial QS (bacterial communication), biofilm formation, and virulence behavior [27], we asked whether PL-18 is also able to simultaneously inhibit NF-*k*B-mediated inflammation. Molecules with dual activity, such as this, may be of increased therapeutic value to address bacterial infections accompanied by severe inflammation.

The NF-*k*B inhibitory activity of PL-18 was determined in noncytotoxic conditions. As previously reported, the cytotoxic potency and ROS formation of PL were correlated to the double bond in the PL alkaloid ring [24]. By contrast, when the methoxy groups were replaced by hydroxyls in PL-18, the molecule acquired antioxidant activity and became less cytotoxic than PL [20]. To determine the potential toxicity of PL-18, in vivo pharmacokinetic and toxicity experiments should be performed.

PL, the parental molecule, is a potent NF-*k*B inhibitor [25]. Here, we showed that its di-hydroxy derivative, PL-18, also significantly inhibited NF-*k*B activation in both L428 and A549 cells. Since L428 lacks I*κ*B, NF-*k*B is constitutively active in these cells, implying that the activity of PL-18 is independent of I*κ*B [35, 36]. We also evaluated the inhibitory activity of PL-18 on A549, which expresses an intact NF-*k*B pathway. To this end, we activated NF-kB in these cells with TNF*α*. PL-18 inhibits NF-*k*B activation in A549 cells in a dose- and time-dependent manner. The total intensity of NF-*k*B during the experiments remains constant and the differences observed were only due to the relative concentration in the nucleus vs. the cytoplasm. This suggests that PL-18 inhibition does not result from NF-*κ*B p65 subunit degradation [37]. When PL-18 was added to TNF*α*-activated cells at different times (before, together, or after TNF*α*), we observed that the best NF-*k*B inhibition was obtained when it was added before or together with TNF*α*. Its addition after TNF*α* activation was much less effective, possibly because PL-18 is better at preventing activation but less so after activation has occurred.

It has been reported that PL, directly interacts with IKK, inhibiting IKK*β* phosphorylation. The unphosphorylated IKK remains constant and is not degraded [38]. By contrast, the WB results showed that in response to PL-18, IKK is degraded in both L428 and A549 cell-lines. This difference indicates that PL-18 and PL have different modes of action regarding the modulation of IKK. Since similar results were obtained with both cell-lines, it shows that the results are most likely significant. Similarly to our results, IKK can be degraded in other systems such as by autophagy [39, 40]. The degradation of IKK could explain the inhibition of NF-*k*B in A549 cells since, in the absence of IKK, I*κ*B will not be phosphorylated and degraded. In L428 cells, where I*κ*B is lacking, IKK directly phosphorylates and activates p65 [41, 42]. PL-18 may interact directly with IKK, resulting in its degradation. Alternatively, PL-18 may induce IKK degradation by upstream molecules.

Infection recognition and the initiation of inflammation are mediated by the production of a variety of inflammatory mediators, including chemokines and cytokines [43]. The cytokine array used here to test if PL-18 affects cytokine secretion showed that PL-18-treated reduced the secretion of the cytokines detected. The control membrane shows MIF, CCL5/RANTES, ICAM-1/CD54, and CXCL12/SDF-1 cytokines. MIF is an important inflammatory factor in infections. Treating with MIF-antibodies in a mouse infection model decreases the sepsis shock caused by LPS [44]. MIF and NF-*k*B pathways are regulatory interconnected [45, 46]. CCL5/RANTES is crucial for recruiting leukocytes [47]. The expression of CCL5/RANTES is regulated by NF-*k*B [48, 49]. The intracellular adhesion molecule ICAM-1/CD54 is also crucial for leukocyte migration and adhesion [50], and its expression is also dependent on NF-*k*B [51, 52]. CXCL12/SDF-1 is secreted by bladder epithelial cells during urinary tract infection [53], it regulates inflammation by NF-*k*B activation [54, 55]. These results suggest that PL-18 is an anti-inflammatory factor with the potential to be further developed.

In conclusion, we have tested the ability of PL-18, the di-hydroxy derivative of PL to inhibit NF-*k*B. We found that PL-18 inhibits NF-*k*B in a dose and a time-dependent manner. Moreover, we determined that the p65 subunit nuclear translocation was inhibited. The use of L428 cells, lacking I*κ*B, directed us to test how PL-18 affects IKK, and found that it is degraded. The originality and uniqueness of PL-18 (not present, to our knowledge, in other commonly used anti-inflammatory or antibiotic drugs) are its dual ability to reduce severe inflammation and, at the same time, to fight persistent bacteria, preventing antibiotic resistance. The combination of both attributes in a single drug gives PL-18 an important advantage in comparison to similar drugs [27]. Thus, PL-18 is a potent candidate molecule to be further developed to treat resistant bacterial infections accompanied by inflammation.

## Data Availability

Data are available from the corresponding author upon request.

## Supporting Information

Supporting Information The supporting file includes Figure S1: The effect of PL-18 on L428 viability and LC50 calculations. Figure S2: The effect of PL-18 on the presence of p65 subunit in A549 cells. Figure S3: Cytokines array membrane at different times of imaging exposure. As well as Table S1: Cytokine spots in membranes exposed to PL-18 or DMSO treated supernatants.
