# Supplementary material for: NF-κB Inhibitory Activity of the Di-Hydroxy Derivative of Piperlongumine (PL-18)
Source: J Immunol Res. 2025 Jan 8;2025:9915695. doi: 10.1155/jimr/9915695 (PMC11735059; doi:10.1155/jimr/9915695)
Supplement: Supporting Information — The supporting file includes Figure S1: The effect of PL-18 on L428 viability and LC50 calculations. Figure S2: The effect of PL-18 on the presence of p65 subunit in A549 cells. Figure S3: Cytokines array membrane at different times of imaging exposure. As well as Table S1: Cytokine spots in membranes exposed to PL-18 or DMSO treated supernatants. [file 9915695.f1.docx]

Supplementary for

**NF-κB Inhibitory Activity of the Di-hydroxy Derivative of Piperlongumine (PL-18)**

Yael Schlichter Kadosh^1+^, Subramani Muthuraman^2+^, Ariel Kushmaro^1, 3, 4^, Rajendran Saravana Kumar^2#^, and Jacob Gopas^5#^.

^1^ Avram and Stella Goldstein-Goren Department of Biotechnology Engineering, Ben Gurion University of the Negev, Beer Sheva 84105, Israel.

^2^ Chemistry Division, Vellore Institute of Technology, Chennai, India.

^3^ The Ilse Katz Center for Nanoscale Science and Technology, Ben Gurion University of the Negev, Beer Sheva 84105, Israel.

^4^ School of Sustainability and Climate Change, Ben Gurion University of the Negev, Beer Sheva 84105, Israel.

^5^ Department of Microbiology, Immunology and Genetics Faculty of Health Sciences, Ben Gurion University of the Negev, Beer Sheva 84105, Israel.

+ Equal contribution.

# Corresponding authors: JG (jacob@bgu.ac.il) and RSK ([sar.org@gmail.com](mailto:sar.org@gmail.com)).

1. PL-18 effect on L428 cells viability and LC_50_ calculation.

The effect of PL-18 on L428 cells viability was tested with trypan blue. PL-18 inhibits L428 cell viability in a dose dependent manner (Supplementary Figure 1 A). There is a linear regression line fit to the PL-18 effect on L428 cell viability (Supplementary Figure 1 B). 50 % is equal to 5 in Probit transformation, thus, to calculate LC_50_, y is equal to 5 (y=1.7796x+3.2485), and the result was 0.9673, and 10 in the power of this resulted in 9.3 µM.


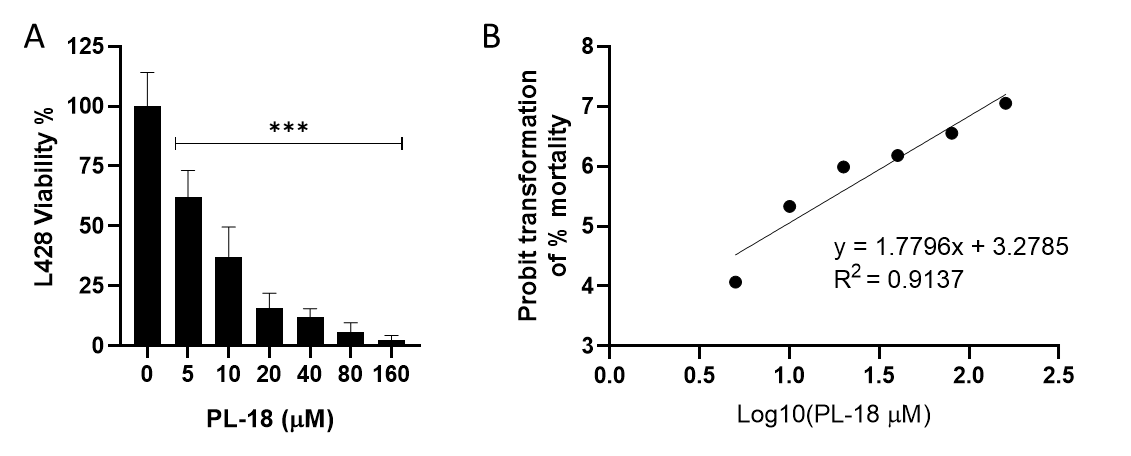


Supplementary Figure 1: The effect of PL-18 on L428 viability and LC_50_ calculations: L428 cell viability was tested with trypan blue. (A) The cell concentration in different PL-18 concentrations was normalized to the control (DMSO) and multiplied by 100 for percentages, this is the viable percentage. (B) To have mortality percentages, the viable percentage is subtracted from 100, and then that is transformed using Probit transformation and plotted against the log of PL-18 concentrations. A linear regression line was generated including equation and R^2^.

2. The presence of subunit p65 in A549 cells in response to PL-18

To measure if the p65 subunit degraded when PL-18 was added, the total intensity of Alexa 488 in A549 cells was determined in the immunofluorescent experiments, dose and time dependency. In both cases, the total intensity was almost constant (Supplementary Figure 2).


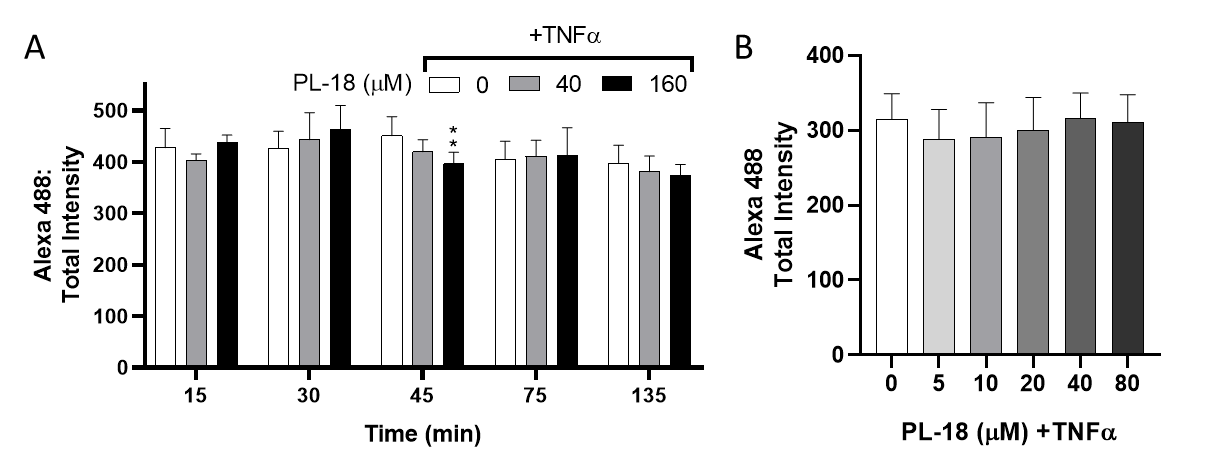


Supplementary Figure 2: The effect of PL-18 on the presence of p65 subunit in A549 cells. (A) A549 cells were co-incubated with two concentrations of PL-18 or vehicle (DMSO) for different time intervals then NF-κB activated with 2.5 ng/mL TNFα for 15 min. (B) A549 were co-incubated with increasing concentration of PL-18 for 4 hrs. and activated with 2.5 ng/mL TNFα for 15 min. For A and B, cells were then fixed, and p65 was immunostained with primary antibody mouse anti p65, then secondary antibody, Alexa 488 conjugated anti-mouse. Imaging was performed at Operetta high throughput fluorescent imaging system and analyzed in the Columbus software. The graphs show the total intensity of Alexa 488, total = nucleus + cytoplasm. For A, Two-way ANOVA, Dunnett’s multiple comparison test N=4, duplicates in two independent experiments. For B, One-way ANOVA, Dunnett’s multiple comparison test, all comparisons were insignificant, N=6, duplicates in three independent experiments.

3. Cytokines

After 4 hours of incubation with PL-18 or DMSO, several cytokines were detected (Supplementary Table 1). Some appeared only after long exposure and could not be quantified appropriately. Therefore, in the main text, we only showed the results of the short exposure. Here, in comparison, we performed the imaging of both exposure times, short (Supplementary Figure 3 A) and long (Supplementary Figure 3 B). The membrane incubated with PL-18 treated supernatants showed a significant reduction in detected cytokines, which was in line with its inhibitory activity.

Supplementary Table 1: Cytokine spots in membranes exposed to PL-18 or DMSO treated supernatants.

| **Spot’s location: DMSO, 4 hrs.** | **Spot’s location: PL-18 160 µM, 4 hrs.** | **CYTOKINE** |
| --- | --- | --- |
| Reference points A1,2 | | |
| Reference points A19,20 | | |
| C3,4 |  | IL-1ra/IL1F3 |
| D3,4 |  | IL-13 |
| E3,4 | E3,4 | MIF |
| B5,6 |  | CXCL12/SDF-1 |
| D5,6 |  | IL-16 |
| E5,6 |  | Serpin E1/PAI-1 |
| A7,8 |  | MIP-1α/MIP-1β |
| B7,8 |  | G-CSF |
| E7,8 |  | TNFα |
| A9,10 | A9,10 | CCL5/RANTES |
| B11,12 | B11,12 | ICAM-1/CD54 |
| C11,12 |  | IL-6 |
| D11,12 |  | IL-18/IL-1F4 |
| B15,16 |  | IL-1α-IL-1F1 |
| A17,18 |  | CXCL10/IP-10 |
| Reference point E 1,2 | | |


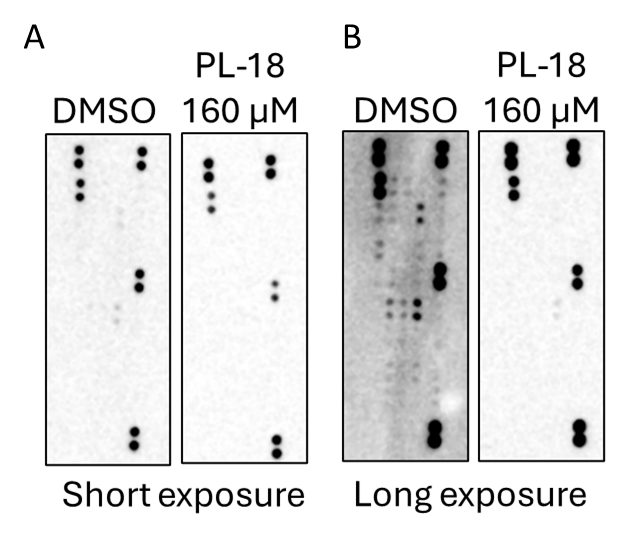


Supplementary Figure 3: Cytokines array membrane at different times of imaging exposure. L428 cells were treated with 160 µM PL-18 or DMSO for 4 hrs., then the supernatants were collected and applied to the human proteome cytokines array. The chemiluminescence developed membranes were analyzed by ImageJ. (A) short and (B) long exposure imaging.
